# Invasive *Burkholderia cepacia* Complex Infections among Persons Who Inject Drugs, Hong Kong, China, 2016–2019

**DOI:** 10.3201/eid2802.210945

**Published:** 2022-02

**Authors:** Kristine Shik Luk, Yat-ming Tsang, Alex Yat-man Ho, Wing-kin To, Ben Kwok-ho Wong, Maureen Mo-lin Wong, Yiu-chung Wong

**Affiliations:** Princess Margaret Hospital, Hong Kong, China (K.S. Luk, Y.M. Tsang, A.Y.M. Ho, W.K. To);; Caritas Medical Centre, Hong Kong (B.K.H. Wong, M.M.L Wong); Yan Chai Hospital, Hong Kong (Y.C. Wong)

**Keywords:** Burkholderia, Burkholderia cepacia complex, disease outbreak, drug users, persons who inject drugs, pulsed-field gel electrophoresis, bacteria, Hong Kong

## Abstract

During March 2016–January 2019, *Burkholderia cepacia* complex (BCC) infection developed in 13 persons who inject drugs (PWID) in Kowloon West Region, Hong Kong, China. Seven cases were infective spondylitis, 2 endocarditis, 2 septic arthritis, 1 intramuscular abscess and bacteremia, and 1 necrotizing fasciitis. Pulsed-field gel electrophoresis revealed that the isolates from 9 patients were clonally related. This clone caused major illness, and 11 of the 13 patients required surgical treatment. Clinicians should be aware of this pathogen and the appropriate broad-spectrum antimicrobial drugs to empirically prescribe for PWID with this life-threatening infection. Close collaboration among public health authorities, outreach social workers, and methadone clinics is essential for timely prevention and control of outbreaks in the PWID population.

*Burkholderia cepacia* complex (BCC) is a ubiquitous aerobic gram-negative bacillus composed of >20 phylogenetically closely related species (*1*). BCC is commonly found in water, soil, and plants and was the cause of onion rot in the 1950s, when it was first described (*2*). It is a rapidly growing bacterium that can survive for a long time in harsh environments. Studies have demonstrated that it is capable of living for >1 year in a 10% iodine solution and can use penicillin as its only energy source (*3*,*4*).

The bacterium’s large genome, twice the size of that of *Escherichia coli*, enables its tremendous adaptability and inherent resistance to multiple antimicrobial drugs (*5*). Its outer membrane is 10 times less permeable that that of *E. coli*, resulting in intrinsic resistance to aminoglycosides and colistin (*6*). The arrangement of circular replicons of transposable elements further enables frequent recombination events. BCC resistance to β-lactams has developed by means of an inducible chromosomal β-lactamase and altered penicillin-binding proteins. Antimicrobial efflux pumps can also lead to resistance to trimethoprim, chloramphenicol, and fluoroquinolones (*6*).

BCC virulence is low, and it generally does not cause illness in immunocompetent persons. Although BCC mainly causes opportunistic infection in patients with cystic fibrosis and chronic granulomatous disease, it has been detected in immunocompromised persons and persons who inject drugs (PWID) who have bacteremia, endocarditis, septic arthritis, or infective spondylitis (*7**–**11*)*.* Outbreaks of nosocomial infection resulting from contaminated heparin and povidone iodine solutions have also been reported (*12*,*13*). The pathogen has been isolated from several medical products such as intravenous fluids, dialysis fluids, ultrasonography gels, nebulizers, thermometers, and tap water (*11*,*14*–*17*).

In October 2018, the infection control officer of Caritas Medical Centre, Kowloon West Region, reported to the Centre of Health Protection, Department of Health, Hong Kong, China, that during March 2017–September 2018, invasive infection caused by a clonal strain of BCC was diagnosed for 8 PWID. We describe the clinical features of patients involved with this unusual outbreak, which may have resulted from external contamination of a batch of drugs or drug paraphernalia. The study protocol was approved by the Kowloon West Cluster Clinical Research Ethics Committee, Hospital Authority.

## Methods

### Clinical Setting

Four regional hospitals (total 3,431 beds) serve an estimated population of 1.4 million in Kowloon West Region, accounting for 19% of the Hong Kong population. We used culture records of the regional microbiology laboratory to identify cases of BCC infection from January 1, 2016, through June 30, 2019. We included in our study PWID patients experiencing their first episode of BCC invasive infection.

Clinical data retrieved from medical charts included patient demographics, underlying diseases, type of abused drugs, signs/symptoms and their duration, sources of infection, recent medical procedures, neurologic status classified by the American Spinal Injury Association impairment scale (https://asia-spinalinjury.org), length of hospital stay, antimicrobial therapy, and surgical treatment. Empiric antimicrobial therapies were defined as antimicrobial agents used before the availability of culture and susceptibility results, and definitive therapies were defined as those used after. We also included leukocyte counts, erythrocyte sedimentation rates, C-reactive protein levels, microbiology results (blood culture, pus aspirate, tissue, or wound swab samples), and radiology findings. The main outcome measures were 7- and 30-day mortality as well as clinical responses at 72 hours after the start of definitive antimicrobial therapy, which included resolution of fever, leukocytosis, and signs of infection.

### Microbiological Studies and Genotyping

BCC was isolated on horse blood agar and identified by matrix-assisted laser desorption/ionization time-of-flight mass spectrometry (MALDI Biotyper; Bruker Daltonics, https://www.bruker.com). We performed antimicrobial susceptibility testing of ceftazidime, levofloxacin, meropenem, minocycline, and trimethoprim/sulfamethoxazole according to the guidelines set by the Clinical and Laboratory Standards Institute (*18*). Because whole-genome sequencing was not available in our laboratory, we determined the clonal relatedness of BCC by using pulsed-field gel electrophoresis (PFGE) of DNA digested with *SpeI* endonuclease (New England BioLabs, https://www.neb.com) as previously described (*19*). For controls, we used 7 archived outbreak-unrelated BCC isolates. We analyzed digitalized gel images by using BioNumerics version 7.0 (Applied Maths, https://www.bionumerics.com). We set banding matching tolerance at 1%. We performed cluster analysis by using the unweighted pair-group method with arithmetic averages based on Dice coefficients to quantify similarities. For PFGE interpretation, we applied the criteria described by Tenover et al. and considered patterns to be closely related or indistinguishable if similarity was >80% (*20*).

## Results

We identified 13 PWID who had BCC invasive infection during March 2016–January 2019, of which 12 were admitted to Caritas Medical Centre and 1 was admitted to Yan Chai Hospital, Kowloon West Region. All PWID used heroin, 11 (84.6%) were men, 11 had hepatitis C, and 4 had a history of deep vein thrombosis (Table 1). The duration of signs/symptoms ranged from 1 to 60 days (median 3 days). Three patients had fever (>38°C), and 7 had spondylodiscitis. 

**Table 1:** Clinical features of invasive infections caused by *Burkholderia cepacia* complex in persons who inject drugs, Hong Kong, China, 2016–2019

**Patient no.**	**Age, y/sex**	**Underlying conditions**	**Symptoms (duration, d)**	**Body temperature, °F (°C)**	ASIA impairment scale*	**Infection type**
**1**	51/F	Diabetes mellitus, hepatitis C	Back pain lower limb numbness and weakness (3)	98.2 (36.8)	D	T11/12 spondylodiscitis with cord compression
**2**	66/M	Hepatitis C	Back pain (20)	97.9 (36.6)	E	L4/5 spondylodiscitis
**3**	66/M	Hepatocellular carcinoma, hepatitis C	Right upper limb numbness and weakness (60)	98.8 (37.1)	D	C5/6, C6/7 spondylodiscitis with prevertebral abscess
**4**	65/M	Deep vein thrombosis, pseudo-aneurysm, right above-knee amputation, hepatitis C	Reduced general condition, dizziness and vomiting (1)	99.3 (37.4)	NA	Endocarditis and septic emboli in lungs
**5**	60/M	Schizophrenia, deep vein thrombosis, pseudo-aneurysm, hepatitis C	Fever, right leg pain and redness (1)	103.1 (39.5)	NA	Intramuscular abscess and bacteremia†
**6**	59/M	Gout	Back pain and bilateral foot numbness (7)	98.4 (36.9)	E	L5/S1 spondylodiscitis
**7**	51/F	Psoas abscess, deep vein thrombosis, fractured right hip with open fixation, hepatitis C	Right hip pain, not able to bear weight (7)	99.0 (37.2)	NA	Right hip prosthetic joint infection and osteomyelitis
**8**	69/M	Asthma, infective spondylodiscitis	Right knee pain (3)	101.1 (38.4)	NA	Right knee septic arthritis and intramuscular abscess†
**9**	62/M	Stroke, hepatitis B, hepatitis C	Fever, back pain, right lower limb numbness, and tingling sensation (4)	99.1 (37.3)	E	L3/4, L5/S1 spondylodiscitis
**10**	64/M	Stroke, deep vein thrombosis, infective cervical spondylitis, hepatitis C	Back pain with radiation to right lateral thigh and weakness (3)	99.3 (37.4)	D	L2/3 spondylodiscitis
**11**	46/M	Esophageal cancer, hepatitis C	Fever, drowsiness, cough, dyspnea (1)	103.3 (39.6)	NA	Endocarditis and septic emboli in brain†
**12**	66/M	Infective endocarditis, hepatitis C	Back pain and lower limb weakness (14)	98.2 (36.8)	D	L1/2 spondylodiscitis with cord compression
**13**	55/M	Hepatitis C	Left knee and thigh pain (2)	96.8 (36)	NA	Left thigh intramuscular abscess and necrotizing fasciitis†

*ASIA impairment scale: A = complete (no motor or sensory function in S4–S5); B = incomplete (sensory function below neurologic level and in S4–S5, no motor function below neurologic level); C = incomplete (motor function is preserved below neurologic level, and more than half of the key muscle groups below neurologic level have a muscle grade <3); D = incomplete (motor function is preserved below neurologic level, and at least half of the key muscle groups below neurologic level have a muscle grade 3); E = normal. ASIA, American Spinal Injury Association; NA, not applicable. **†Polymicrobial infection: *Arcanobacterium haemolyticum* in abscess aspirate of patient 5; *Streptococcus pneumoniae, Streptococcus constellatus*, and *Bacteroides fragilis* in joint fluid from patient 8; *Staphylococcus aureus* and *Enterococcus faecalis* in blood of patient 11; and group G *Streptococcus*, *Peptoniphilus harei*, and *Finegoldia magna* in abscess aspirate from patient 13. **

Concerning antimicrobial therapy, most (n = 10) patients were empirically given amoxicillin/clavulanate (Table 2). All BCC isolates were susceptible to ceftazidime, levofloxacin, meropenem, minocycline, and trimethoprim/sulfamethoxazole. For definitive therapy, 6 patients were given trimethoprim/sulfamethoxazole as combination treatment with levofloxacin (n = 3), ceftazidime (n = 2), and meropenem (n = 1). Among patients with spondylodiscitis, 5 had lumbar spine involvement, and all except 1 had undergone surgery. Four patients had polymicrobial infection. All patients showed good response to treatment. One patient had a relapse of BCC spondylodiscitis 5 months after a 6-week course of meropenem and oral trimethroprim/sulfamethoxazole and subsequently underwent spinal fusion. Two patients died, one of hepatocellular carcinoma and the other of an unknown cause (certified dead at the emergency department 1 day after discharge). Neither patient underwent autopsy. Attempts to contact the surviving patients regarding their drug use behaviors were unsuccessful.

**Table 2:** Laboratory and microbiological findings, treatments, and outcomes of invasive infections caused by *Burkholderia cepacia* complex in persons who inject drugs, Hong Kong, China, 2016–2019*

Variable	Finding
Median leukocytes, × 10^9^ cells/L (range)	9.2 (3.94–24.7)
Median ESR, mm/h (range)	79.5 (43 to >120)
Median CRP, mg/L (range)	68 (21 to >294)
Empirical antimicrobial therapy (no. patients)	Amoxicillin/clavulanate (10); cloxacillin (3); vancomycin (2); ampicillin, cefoperazone/sulbactam, gentamicin, piperacillin/tazobactam (1)
Definitive antimicrobial therapy (no. patients)	Trimethoprim/sulfamethoxazole (6); ceftazidime (5); levofloxacin (5); meropenem (2); minocycline, piperacillin/tazobactam (1)
Median duration of antimicrobial therapy (range), wk	6 (1–12)
Surgery (no. patients)	Vertebral disk excision (3), incision and drainage (1), spinal fusion† (3), joint arthrotomy (2), excisional arthroplasty (1)
Positive culture (no. patients)	Bone (5), blood† (4), intervertebral disk (3), abscess (2), joint fluid (1)
Median length of stay (range), d	43 (11 – 97)
Death (d after first visit)	Patient 3 (124); patient 4 (13)

*CRP, C-reactive protein; ESR, erythrocyte sedimentation rate. †Patient 2 had a relapse of *Burkholderia cepacia* complex infective spondylitis 5 mo after receiving meropenem and oral trimethoprim/sulfamethoxazole for 6 wk; spinal fusion was performed during the relapse episode.

**Figure 1:**
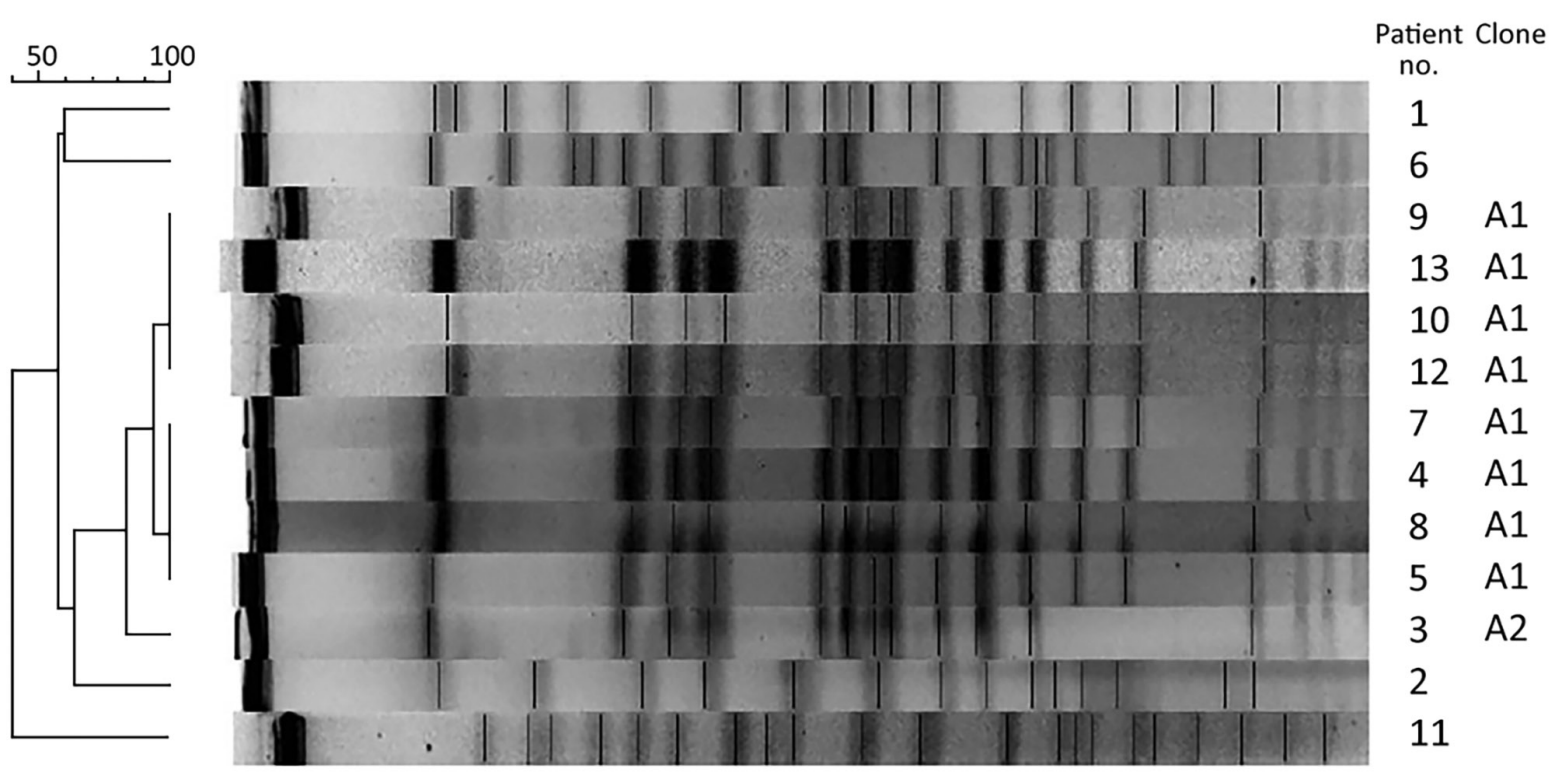
Pulsed-field gel electrophoresis of *Burkholderia cepacia* complex causing invasive infection in 13 persons who inject drugs, Hong Kong, China, 2016–2019.

**Figure 2:**
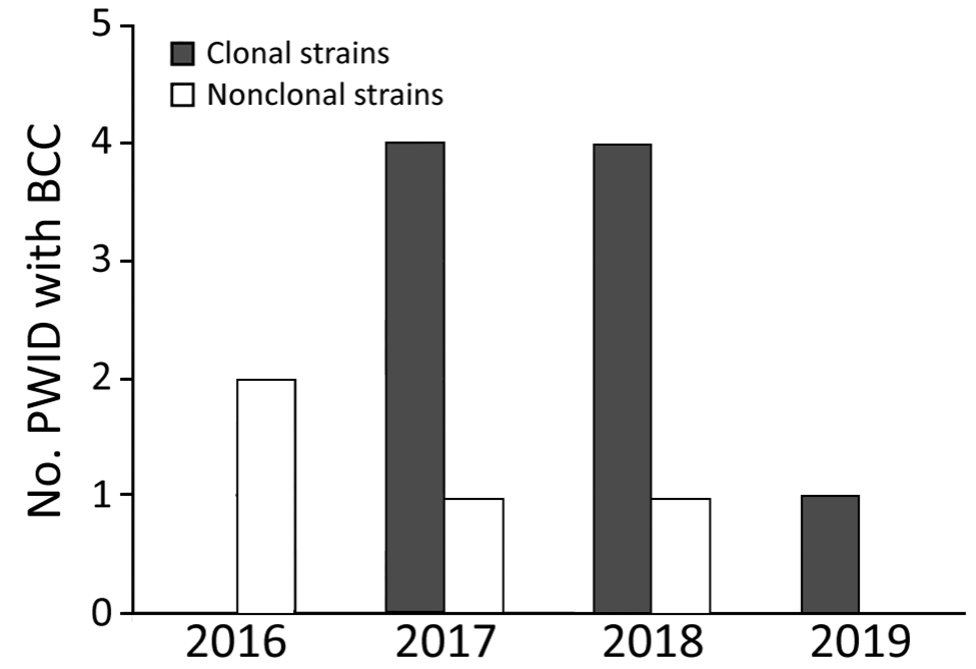
Number of PWID with newly detected BCC invasive infection, by clonal types per year, Hong Kong, China, 2016 through June 30, 2019. PWID, persons who inject drugs; BCC, *Burkholderia cepacia* complex.

PFGE analysis of the isolates showed that 9 of the 13 invasive infections were caused by the same clone (Figure 1); banding patterns were identical for 8 isolates. All patients except 1 lived in the Shum Shui Po District, which has a total area of 9.48 km^2^, within the Kowloon West Region. A marked increase of the clonal isolates was noted during 2017–2018 (Figure 2).

## Discussion

In 2018, a reported 3,579 persons in Hong Kong injected heroin (*21*). As a result of hematogenous seeding, drug injection is a substantial risk factor for musculoskeletal infections, including septic arthritis and osteomyelitis of the spine and infective endocarditis (*22*). The common responsible organisms are *Staphylococcus aureus*, *Streptococcus* spp., and occasionally *Pseudomonas aeruginosa *(*22*)*.* Other outbreaks of life-threatening infections caused by *Clostridium* spp. and *Bacillus anthracis* have also been associated with use of contaminated heroin (*22*,*23*). On the contrary, the literature has rarely reported BCC as the culprit of drug-injection–associated musculoskeletal infections or infective endocarditis (*24*–*27*). Our report of a common-source outbreak of BCC invasive infection among PWID, in a highly localized geographic area of Hong Kong and supported by PFGE, is unusual.

The mechanism of spread of the epidemic strain of BCC among PWID was unclear because neither microbiological investigation regarding the possible contaminated batch of heroin nor environmental sampling of the injection gallery or drug distribution site was conducted by the local public health agency. BCC is commonly found in the environment instead of the commensal flora. In addition, there was a strong association between the outbreak and the place of residence of PWID. The mode of transmission was possibly through the drug, drug adulterants, or drug paraphernalia. Bacterial contaminants, including *P. aeruginosa*, were identified in 40% of seized drug samples (*28*). *P. aeruginosa* contamination of syringes causing an outbreak of infective endocarditis has also been documented (*29*). Most patients in our study had hepatitis C virus infection, and sharing paraphernalia (e.g., needles) was believed to be frequent. Needle sharing could increase transmission of an epidemic strain. A recent polyclonal BCC outbreak in peritoneal dialysis patients in Hong Kong was caused by contaminated aqueous chlorhexidine (*30*); however, PFGE typing of the strains from the patients in our study and the strains isolated in the aqueous chlorhexidine suggested that they were unrelated (K.S. Luk, unpub. data). The preponderance of BCC in the outbreak we report could result from its environmental survival advantage and intrinsic resistance to multiple disinfectants and antimicrobials. The outbreak ended after the Centre of Health Protection issued press releases (*31*), and the health advice to inject only with single-use disposable needles was strengthened at methadone clinics and by outreach social workers.

Invasive infections caused by BCC are rarely reported and result in considerable illness. The high infection rate may also be increased by contributions from malnutrition, concurrent chronic viral hepatitis, and lymphocyte opiate receptor–mediated natural killer T-lymphocyte inactivation (*22*). The most commonly used antimicrobial option is trimethoprim/sulfamethoxazole. Because of resistance to multiple antimicrobial drugs, successful treatment usually requires combinations of drugs and surgery. Drug combinations that have demonstrated synergy in vitro include trimethoprim/sulfamethoxazole and ceftazidime, ciprofloxacin and carbapenem, and ceftazidime and ciprofloxacin (*32*).

The most frequent invasive infection in our series was spondylodiscitis. Arterial seeding is apt to involve the vascular intervertebral disk and adjoining vertebral end plates. Most frequently involved was the lumbar area (5 patients), followed by the thoracic (1 patient) and cervical (1 patient) areas. BCC spondylodiscitis is a rare entity; the medical literature has reported only 6 cases, 3 of which were in patients with the risk factor of being PWID (*11*,*25*,*26*). Pyogenic osteomyelitis after rhinoplasty has also been described; other affected persons were a healthy elderly farmer and a patient after a fall and intramuscular procedure (*33*–*35*). Similar to the reported cases, patients in our study did not have fever, and symptom onset duration varied from 1 day to 2 months. The subtle exacerbation of axial pain might contribute to delayed diagnosis, as reflected by most (5 of 7) patients for whom abnormal findings were seen on plain vertebral radiographs. All patients had preserved sensory function, and 4 had mild limb weakness. Of note, 5 patients had a normal leukocyte count at the time of admission, and all had elevated inflammatory markers. A history of injection drug use associated with axial pain should alert clinicians to the possibility of spinal infections, despite absence of fever, abnormal leukocyte count, or physical examination or radiographic findings. Further diagnostic workup to exclude spinal infection, including magnetic resonance imaging with and without contrast, should be performed. In addition, empiric prescription of antimicrobial drugs should be withheld to increase the chance of isolating an organism, which is advantageous for a successful outcome. In this case, BCC was resistant to commonly prescribed antimicrobial drugs such as amoxicillin/clavulanate, so BCC was successfully isolated from vertebral tissue (3 patients). intervertebral disk (3 patients), and blood culture (1 patient). Because bacteria were positively identified in blood culture, patient 6 did not undergo further biopsy or surgical treatment. He recovered after 6 weeks of treatment with ceftazidime and oral trimethoprim/sulfamethoxazole. 

Three patients underwent spinal fusion because of infection relapse (patient 2), cord compression (patient 1), and spinal instability (patient 3). Of 3 patients with early infection, discectomy and oral levofloxacin for 6 months resulted in cure. Of the 13 patients, outcomes were good for 12, but 1 patient (patient 3) died of metastatic hepatocellular carcinoma 4 months after initial visit. 

Oral administration of antimicrobial drugs has the advantage of avoiding difficult intravenous access and the need for prolonged hospitalization. Nevertheless, parenteral therapy for <4 weeks is associated with a 25% relapse rate, and use of oral antimicrobial drugs should be reserved only for patients with early disease under close monitoring of clinical response (*36*).

Endocarditis caused by BCC was more commonly reported for patients with prosthetic valves (*37*) and for patients with a history of injection drug use and native valve involvement (*38*). Patient 3 exhibited the clinical features of right-sided endocarditis. The diagnosis was not suspected, and ceftazidime was prescribed for only 8 days. The patient’s death shortly after discharge may be associated with the endocarditis, although no autopsy was performed. Patient 11 had polymicrobial infection (BCC, *S. aureus*, and *Enterococcus faecalis*) of the mitral valve and cerebral septic emboli. Treatment with ampicillin, cloxacillin, and meropenem was successful without valve replacement, despite the relatively large size (2.0 × 1.5 cm) of the vegetation. Successful outcome after antimicrobial treatment has been reported for only a few cases (*39*,*40*). An endovascular source should be investigated for all PWID with bacteremia, and early treatment may substantially improve patient outcomes.

Only 5 previous reports have described BCC isolation from joint fluid culture, affecting the ankle, knee, shoulder, and hip. Predisposing factors included history of intra-articular steroid injection, weakened immunity because of T-cell lymphoma or premature birth, and physical trauma (*10*,*41*–*45*). Patient 7 had a right prosthetic hip joint infection 6 weeks after undergoing screw fixation of the proximal femur; the source of the infection was speculated to be hematologic seeding. The patient had glucose-6-phosphate dehydrogenase deficiency, and trimethoprim/sulfamethoxazole was not given. Infection was well controlled with excisional arthroplasty and administration of ceftazidime for 11 weeks. The implanted device was removed because of severe contracture and dislocation of hip cement spacer. 

Patient 8 had an intramuscular abscess of the right thigh and polymicrobial septic arthritis (BCC, *Streptococcus pneumoniae, Streptococcus constellatus, Bacteriodes fragilis*) after having injected drug into the right thigh. The injection site was probably contaminated. Treatment consisted of 4 weeks of oral levofloxacin, minocycline, and metronidazole, as well as multiple operations including incision and drainage of the thigh abscess, arthroscopy, and arthrotomy. The clinical course and inferior outcome were also reported for a patient with polymicrobial septic arthritis after a trauma-induced wound had been contaminated with dirt and soil (*43*).

Two patients had polymicrobial intramuscular abscesses of the thigh after drug injection. For patient 5, BCC was also isolated in blood culture; infection was cured after surgical drainage and debridement and 2 weeks of ceftazidime. Patient 13 experienced septic shock, and an intramuscular abscess (BCC, group G* Streptococcus*, *Peptoniphilus harei*, *Finegoldia magna*) was complicated by early necrotizing fasciitis. The patient required ventilator and vasopressor support and stayed in an intensive care unit for 7 weeks. He responded well after 2 weeks of piperacillin/tazobactam, multiple debridements, left knee arthrotomy (group G* Streptococcus* in joint fluid), and skin grafting. It is worth considering broad-spectrum antimicrobial drugs to cover gram-positive, nonfermentative gram-negative, and anaerobic bacteria for PWID who have severe musculoskeletal infections possibly caused by injection-site contamination.

A limitation of our study is that we failed to contact the surviving patients regarding their drug-use behaviors, and the local health agency did not initiate an investigation including microbiological testing of confiscated heroin or environmental sampling of the injection gallery or drug distribution site. Therefore, we were not certain about the mode of transmission. Also, we did not conduct a prospective epidemiologic study of BCC among PWID, and we may have underestimated the scale of the outbreak because patients with mild infection might not seek hospital care and specimens would not be obtained for culture. The baseline incidence of invasive BCC infection in PWID was <1 case/year, and we believe that the predominance of clonal strains during 2017–2019 represented a significant outbreak. Last, whole-genome sequencing is not readily available in our laboratory, and the resolution of PFGE may not be enough for a large-scale outbreak. Nevertheless, there is no well-established cutoff for the number of single-nucleotide polymorphisms of BCC to be classified as a cluster, and it could be quite variable (*26*). PFGE consists of genomic information that was considered to provide sufficient information for evaluating this focused outbreak (*46*–*49*).

In conclusion, this clonal epidemic of BCC invasive infections among PWID in a Hong Kong region was rare. BCC isolated from sterile sites may be dismissed as sporadic, especially for patients with the risk factor of injection drug use. Molecular typing enables the timely identification of an outbreak and contributes to the investigation and control measures and should be routinely performed for BCC isolated from sterile sites (e.g., blood, pus aspirate, joint fluid, tissue, bone) in special patient groups, such as PWID. The finding of clonal isolates from multiple patients within a geographic area should prompt further investigation by public health agencies.

This outbreak caused severe illness among affected patients. If healthcare providers are alert to invasive infection caused by this unusual, multidrug-resistant organism in PWID, they can empirically prescribe appropriate broad spectrum antimicrobial drugs for PWID with life-threatening infection. At the other end of spectrum, the clinical appearance of patients may be subtle, and further diagnostic workups should be performed so as not to miss any debilitating infection.
